# Combined anti-C1-INH and radiotherapy against glioblastoma

**DOI:** 10.1186/s12885-023-10583-1

**Published:** 2023-01-30

**Authors:** Emma Liljedahl, Elise Konradsson, Emma Gustafsson, Karolina Förnvik Jonsson, Jill K. Olofsson, Kurt Osther, Crister Ceberg, Henrietta Nittby Redebrandt

**Affiliations:** 1 The Rausing Laboratory, Division of Neurosurgery, Department of Clinical Sciences, Lund University, BMC D10, 221 84 Lund, Sweden; 2 Medical Radiation Physics, Department of Clinical Sciences, Lund University, Lund, Sweden; 3 Department for Geosciences and Natural Resource Management, University of Copenhagen, Copenhagen, Denmark; 4 Department of Neurosurgery, Skåne University Hospital, Lund, Sweden

**Keywords:** Glioblastoma, Radiotherapy, Complement system

## Abstract

**Background:**

A more effective immune response against glioblastoma is needed in order to achieve better tumor control. Radiotherapy can induce anti-tumor mediated immune reactions, in addition to its dose response effects. The complement system can function as a bridge between innate and adaptive immune responses. Combining radiotherapy and complement activating therapy is theoretically interesting.

**Methods:**

Radiotherapy at 8 Gy × 2 was combined with treatment against C1-inhibitor (C1-INH), a potent inhibitor of activation of the classical pathway of the complement system. Anti-C1-INH was delivered as intratumoral injections. Fully immunocompetent Fischer 344 rats with NS1 glioblastoma tumors were treated. Survival was monitored as primary outcome. Models with either intracranial or subcutaneous tumors were evaluated separately.

**Results:**

In the intracranial setting, irradiation could prolong survival, but there was no additional survival gain as a result of anti-C1-INH treatment. In animals with subcutaneous tumors, combined radio-immunotherapy with anti-C1-INH and irradiation at 8 Gy × 2 significantly prolonged survival compared to control animals, whereas irradiation or anti-C1-INH treatment as single therapies did not lead to significantly increased survival compared to control animals.

**Conclusions:**

Anti-C1-INH treatment could improve the efficacy of irradiation delivered at sub-therapeutic doses and delay tumor growth in the subcutaneous tumor microenvironment. In the intracranial setting, the doses of anti-C1-INH were not enough to achieve any survival effect in the present setting.

**Supplementary Information:**

The online version contains supplementary material available at 10.1186/s12885-023-10583-1.

## Background

Glioblastoma (GBM) is the most common form of high-grade malignant brain tumor. Current treatment regimens involve maximal safe surgical resection, chemo-radiotherapy, and adjuvant chemotherapy. However, there are only a few effective treatment options for patients with GBM, and still, the outcome is poor, with a median survival of around 12–15 months only, in well selected patients included in clinical studies with conventional therapy [1].

The complement system serves as a major component of innate immunity, and its interaction among complement activation products and cell surface receptors also contributes to activation of the adaptive immune response [2]. The complement system is involved in maintaining homeostasis by detecting and responding to pathogens and altered self. It can be activated by different molecular structures, including antibodies, which initiate a proteolytic cascade marking cells for destruction. Complement is activated through three different pathways: the classical, the lectin mediated, and the alternative pathway [2]. The classical pathway activation is inhibited by C1-INH (C1-inhibitor). C1 consists of subcomponents C1q, C1r and C1s. Binding of C1q to antibody subverts the control of C1-INH by causing dissociation of C1-INH from pro-C1 and allowing autocatalytic cleavage to proceed. At some point after C1 activation, C1-INH binds covalently to the active sites on C1r and C1s, inactivating their catalytic function and dissociating them from C1q. C1-INH binding to C1r and C1s is irreversible; it prevents cleavage of C4 and thereby controls the initial amplification step of classical-pathway activation [3]. Thus, C1-INH binding to C1r and C1s acts as an efficient inhibitor of the classical pathway of the complement system [4, 5]. C1-INH also limits the activity of MASP-2 and several proteases of the coagulation/anticoagulation system, including factor XI, factor XII, plasma kallikrein, plasmin, and tissue plasminogen activator [3, 6]. C1-INH regulates the formation of bradykinin. The genetic deficiency of the C1-INH is responsible for hereditary angioedema (HAE), which is a disease transmitted as an autosomal dominant trait [4]. More recently, it has been suggested that treatment with C1-INH might reduce the effects of brain edema due to traumatic brain injury as observed in an experimental rat model [7]. In a pre-clinical setting, C1-INH was administered intravenously in the same session as a traumatic injury was inflicted upon rats, and after 48 h the brains were weighted before and after heating in order to establish the degree of edema. C3a levels, which can reflect complement activation, in the brains were reduced in animals treated with C1-INH [7]. Interestingly, administration of C1-INH also seems to inhibit hyper-acute rejection in transplantation settings in animal models, whereas little is known in the human settings [6].

The role of the complement system in cancer is complex and context dependent, where a low degree of activation seems to promote tumor progression while potent activation has antitumor effects [8]. Deposits of complement components have been documented in several human tumors suggesting a potential involvement of the complement system in tumor immune surveillance. The lectin pathway has also been implicated in complement activation on glioma cells which express, like many other malignant cells, high mannose glycopeptides that bind MBL and trigger consumption of C4 and C3, but this reaction fails to induce cell lysis [9].

Treatment with monoclonal antibodies against tumor associated antigens can lead to complement dependent cytotoxicity. This is mediated by C1 activation, C3b deposition on cells, C5 cleavage, and C5b formation. C5b is part of the C5b-C6-C7-C8-C9 complex, also known as the membrane attack complex (MAC), which eventually leads to cell lysis [10]. The efficacy of many antibody based immunotherapies is compromised by regulators of the complement system, whose role is to protect the host from unspecific complement activation [10]. CD59 inhibits MAC formation by binding to C8 and C9, and is highly expressed in many cancer forms [10]. Blocking CD59 resulted in improved treatment efficacy in studies on lung cancer and lymphoma. Another example is PTXA3, that interacts with C1q and factor H to modulate complement activation [8]. In PTXA3 deficient mouse models, a constant state of complement mediated inflammation was present, favoring skin carcinoma; whereas high levels of PTXA3 in human cancer was associated with shorter survival [8].

Our previous research has demonstrated that C1-INH is upregulated in glioblastoma, both in human tumors and in rat tumor cells [11]. Treatment with anti C1-INH in relatively large doses could increase survival in fully immunocompetent rats with syngeneically inoculated glioblastoma [12]. GM-CSF and IL-1b were decreased in serum from animals treated with anti-C1-INH, but the exact mechanistic steps leading to increased survival in the animals has not yet been demonstrated [12]. However, it is known from other studies that any damage to the brain can increase GM-CSF [13]. GM-CSF seems to have dual roles in the context of cancer, stimulating dendritic cells and macrophage activity for example, but also promoting tumor growth and metastases [14].

Radiotherapy is generally seen as a local treatment, but it is now well established that radiation also has immunomodulatory effects, which can be explored in combination with immunotherapy [15, 16]. A direct lethal effect on cancer cells by irradiation is mainly caused by DNA damage [17]. A more indirect damage is caused by the production of free radicals, which can lead to both a sub-lethal and lethal damage [17]. Immune mediated effects of irradiation can be caused by modulation of the tumor microenvironment and modification of tumor phenotypes [17]. Cell death induced by irradiation also leads to the release of cytokines, chemokines and tumor antigens.

According to previous research by our group [16] and others [17–20], radiotherapy delivered at optimal doses and fractions can induce an effective immune response, which seems to function in synergy with immunotherapy, potentially also in the intracerebral setting. Delivery of radiotherapy with multiple fractions has been shown to decrease lymphocyte count in circulating blood. Decreasing the fractions and increasing the dose in each fraction, on the other hand, has been suggested to lead to reduced lymphopenia [18]. Pre-clinical studies have demonstrated that hypo-fractioned high doses result in increased pro-immunogenic effect [17, 21]. For example, in experimental breast cancer models, a distant anti-tumor effect outside the irradiation field, the so called abscopal effect, could be demonstrated when irradiation was fractioned and combined with immunotherapy [21]. More specifically, it has been demonstrated that radiotherapy can promote T-cell specific immune cell response [20, 22]. The efficacy within the intracranial setting remains to be demonstrated further.

For many cancer diagnoses, clinical trials combining immunotherapy and radiotherapy are already well under way [23]. The balance between these two components, and thus the net anti-tumoral effect, appears to depend on the time, dose and fractionation of the radiation treatment [15, 24]. Interestingly, radiotherapy has been shown to induce a transient complement activation, both in murine and human tumors, at least through C3a and C5a [25]. Treatment with Dexamethasone reduced the local complement activation and the effect of radiotherapy [25]. It was concluded that certain complement components might be essential for tumor-specific immunity due to radiotherapy. Interestingly, also C5aR1 and C3aR1 seems to play a role in in T-cell induction, where absence of signaling via C5aR1 and C3aR1 lead to a differentiation into FoxP3 + regulatory T (Treg) cells instead of effectors [26].

In the present study, we explored the efficacy of combining anti-C1-INH antibodies with radiotherapy in two different tumor microenvironments. Specific aims were:

1. Exploring the efficacy of combined radiotherapy and anti-C1-INH antibody treatment in experimental glioblastoma.
2. Comparing efficacy in intracranial versus subcutaneous tumor microenvironments.

The radiation dose of 8 Gy × 2 was based on previous studies, where we theoretically could establish that reduced numbers of fractions and increased dose per fraction would be beneficial in regards to immune system activation [19]. The dose of anti-C1-INH was based on a previous rat study with inoculated syngeneic glioblastoma cells [12], where we could demonstrate increased survival as a result of intratumoral treatment in subcutaneous tumors. The dose, however, was reduced in the present set-up, since we wanted to demonstrate a possible interaction with radiotherapy, and thus aimed at delivering the individual therapies as potentially sub-therapeutic doses and levels.

## Methods

### Ethics statement

This study was approved by the Malmö-Lund Animal Research Ethics Committee (permit ID 5–8-18–02,383/2020). All experiments were performed in accordance with relevant guidelines and regulations. All efforts were made to minimize animal suffering and in accordance with ARRIVE guidelines.

### Glioblastoma cells

The rat glioma cell line NS1 [27] was used in the pre-clinical rat studies done in the present study. NS1 is a GFP positive tumor cell from GFP-positive Fisher 344 rats, developed at our laboratory as previously described [27]. The glioma cells can be syngeneically inoculated into Fischer 344 rats where it generates infiltrative tumors, with perivascular dissemination. The rat glioma cells (NS1) were cultured using RPMI-1640 (Sigma-Aldrich) medium with addition of 1% ml Na-pyruvate, 1% ml HEPES (4-(2-hydroxyethyl)-1-piperazineethanesulfonic acid), 0.1% ml Gentamycin, as well as 10% inactivated fetal calf serum (heated to 56 °C for 30 min), as previously described [27]. Sandwich Elisa done according to the manufacturer’s instructions (MycoProbe R&D Systems) was used to rule out Mycoplasma infection in the cells and supernatant.

### Animals

Fischer 344 rats were used (Fischer Scientific, Germany) for the study. The rats were housed in pairs in rat cages with water and rat chow ad libitum. The criteria for euthanasia included signs of paresis or declined general condition regarding intracranial tumors, and tumors exceeding 30 mm in maximal diameter or ulcers related to tumor growth through the skin regarding subcutaneous tumors. Inoculations were performed under general anesthesia with isoflurane inhalation.

### Intracranial tumors

NS1 cells were prepared for inoculation by removal of the medium and washed gently in PBS. The adherent cells were detached by adding Trypsin (Invitrogen). After addition of medium, cells were counted. The cells were centrifuged at 1200 rpm for 5 min at 4 °C and the supernatant removed. The cell pellet was re-suspended in serum-free medium. A number of 5 000 cells were used for each intracranial inoculation. Intracerebral inoculation was done under isoflurane inhalation anaesthesia using a stereotactic frame and inoculation of cells was done using a 10 µl Hamilton syringe. The cells were injected at a depth of 5 mm from the skull, 2 mm laterally from the sagittal suture, and 1 mm anterior to the coronal suture, on the right side of the cranium. The cranial burr hole was sealed with bone wax, and the incision was closed with absorbable suture. Animals were monitored on a daily basis and euthanized if their symptoms met the criteria defined by the ethics regulations, including impaired general condition and neurological deficits.

### Subcutaneous tumors

NS1 cells were prepared for inoculation by removal of the medium and washed gently with PBS as described above. 50 000 cells were used for each subcutaneous injection. Animals were observed up to day 100 after tumor cell inoculations and were euthanized according to the ethics regulations. The animals were euthanized prior to day 100 if the tumor reached a maximal diameter exceeding 30 mm in accordance with ethics regulations.

### Anti-C1-INH administration

Animals with intracranial tumors were treated with immune therapy administered as intratumoral injections of 0.4 ul anti-C1-INH (6.15 mg/ml) (Covance) on days 0 and 10 using the stereotactic frame as described for tumor inoculations, through the same burr hole and at the same depth. The volume was limited in accordance with the animal ethical permit.

Animals with subcutaneous tumors was treated with 0.1 ml anti-C1-INH (6.15 mg/ml) (Covance), administered by intratumoral injections on days 0, 7 and 14.

### Radiotherapy

Radiotherapy was administered in two fractions consisting of 8 Gy on days 7 and 14 for subcutaneous tumors and days 8 and 12 for intracranial tumors, on a 220 kV preclinical research platform (XenX, XStrahl Inc, Suwanee, GA, USA). Animals were positioned one-by-one in PMMA boxes and irradiated using a circular field with a diameter of 1 cm and a dose rate of 3 Gy/min at a source-to-isocenter distance of 35 cm. The absorbed dose was selected as 2 mm depth for subcutaneous tumors, and 5 mm depth for intracranial tumors The treatment unit was calibrated in accordance with the IAEA TRS-398 reference dosimetry protocol, and the delivered dose was verified by using GafChromic EBT3 film (Ashland Advanced Materials, Bridgewater, NJ, USA).

### Immunofluorescence

In order to verify GFP and C1-INH expression in vitro, cells were cultured for 1–2 days in two-chamber culture slides (Thermo Fisher Scientific) at 37 °C in a humidified 5% CO2 incubator. The medium was then removed, and the cells were fixed in 4% paraformaldehyde. The cells were mounted with Eukitt Quick-hardening mounting medium (Sigma-Aldrich). Affinity purified rabbit anti-rat C1-INH antibodies (Covance) were applied in order to detect coating of rat C1-INH on the cells using secondary Alexa Fluor staining. Hoechst (Thermo Scientific) staining was used for nuclear detection. The cells were photographed using a fluorescence microscope fitted with the appropriate wavelength filters.

### Immunohistochemistry

Extracted tumors were fixated with phosphate-buffered 4% paraformaldehyde. The paraffin embedded tumors were cut into 7 µm slices using a cryostat, mounted on adhesion microscope slides.

Antigen identification was performed on de-paraffinized sections. Sections were submerged in pre-heated (100 °C) citrate buffer (Citrate Buffer, pH 6.0, 10 × , Antigen Retriever, Sigma-Aldrich) for 20 min and washed with PBS before immunohistochemistry.

Detection of CD8, CD4 and FOXP3 was performed using ready-to-use Vectastain ABC kit (Vector Laboratories, CA, USA) in combination with primary antibodies consisting of rabbit anti-CD8 (1:200, Sigma-Aldrich, MSA48-GA), mouse anti-CD4 (1:200, Sigma-Aldrich, SAB4700733), and rabbit anti-FoxP3 (1:200, Antibodies online, ABIN3187942), individually.

Briefly, sections were incubated with normal rabbit or mouse serum diluted to 1:200 in PBS containing 0.25% Triton. Following blocking, the sections were incubated with primary antibodies diluted in PBS containing 0.25% Triton and 1% BSA at 4 °C, overnight and subsequently incubated with secondary antibody and ABC reagent, 30 min each in room temperature. The antigen–antibody complex was visualized using the DAKO Liquid DAB Substrate-Chromogen System (DAKO, CA, USA). All sections were stained with hematoxylin (Mayers HTX, ready-to-use, Histolab) for 2 min. Images were captured using an Olympus VS120-26–096 Virtual Slide Microscope with a × 20 objective using and Olympus VS-ASW 2.9 software.

### Gene analysis

In animals that were euthanized due to intracranial tumor growth, part of the tumor was dissected and frozen in liquid nitrogen.

RNA extraction was performed using RNeasy kit (Qiagen®) according to the manufacturer’s instructions, as previously described by us [28]. Briefly, RNA sequencing was performed at the Center for Translational Genetics, Lund University, Sweden. Data was analyzed in R v.3.6.3 (R core team 2020). Differential gene expressions between groups of samples (tissue or treatment) were assessed using edgeR v. 3.28.1 [29–31]. All genes, where two or more samples had fewer than 10 reads, were removed prior to the analyses, as previously described [28]. The raw read counts were normalized to reads per million (rpm) for each sample. Individual genes were considered expressed different, if there were at least a twofold change between groups of samples and the fold change was significant (*p* < 0.05 after correction for multiple testing) and adjusting for expression levels using a generalized linear model provided in the R package as previously described [28]. Intracranial tumor samples, with sufficient RNA quality to pass initial quality checks, were used for gene expression analysis (*n* = 1 control animals; *n* = 4 treated with RT 8 Gy × 2; *n* = 1 treated with anti-C1-INH; *n* = 1 treated with RT 8 Gy × 2 + anti-C1-INH).

### Statistics

SPSS was used for statistical evaluations, except the gene analysis, where R was used as described above. Normality was assessed using Shapiro–Wilk’s test and visual inspection of normality plots. Kruskal–Wallis test was performed on non-parametric data, and Bonferroni corrections were applied in cases of multiple hypotheses testing. ANOVA test was performed on parametric data, and Bonferroni corrections were applied in cases of multiple hypotheses testing. A *p*-value < 0.05 was used for significance.

## Results

### Demonstration of anti-C1-INH binding of glioblastoma cells in vitro

**Fig. 1:**
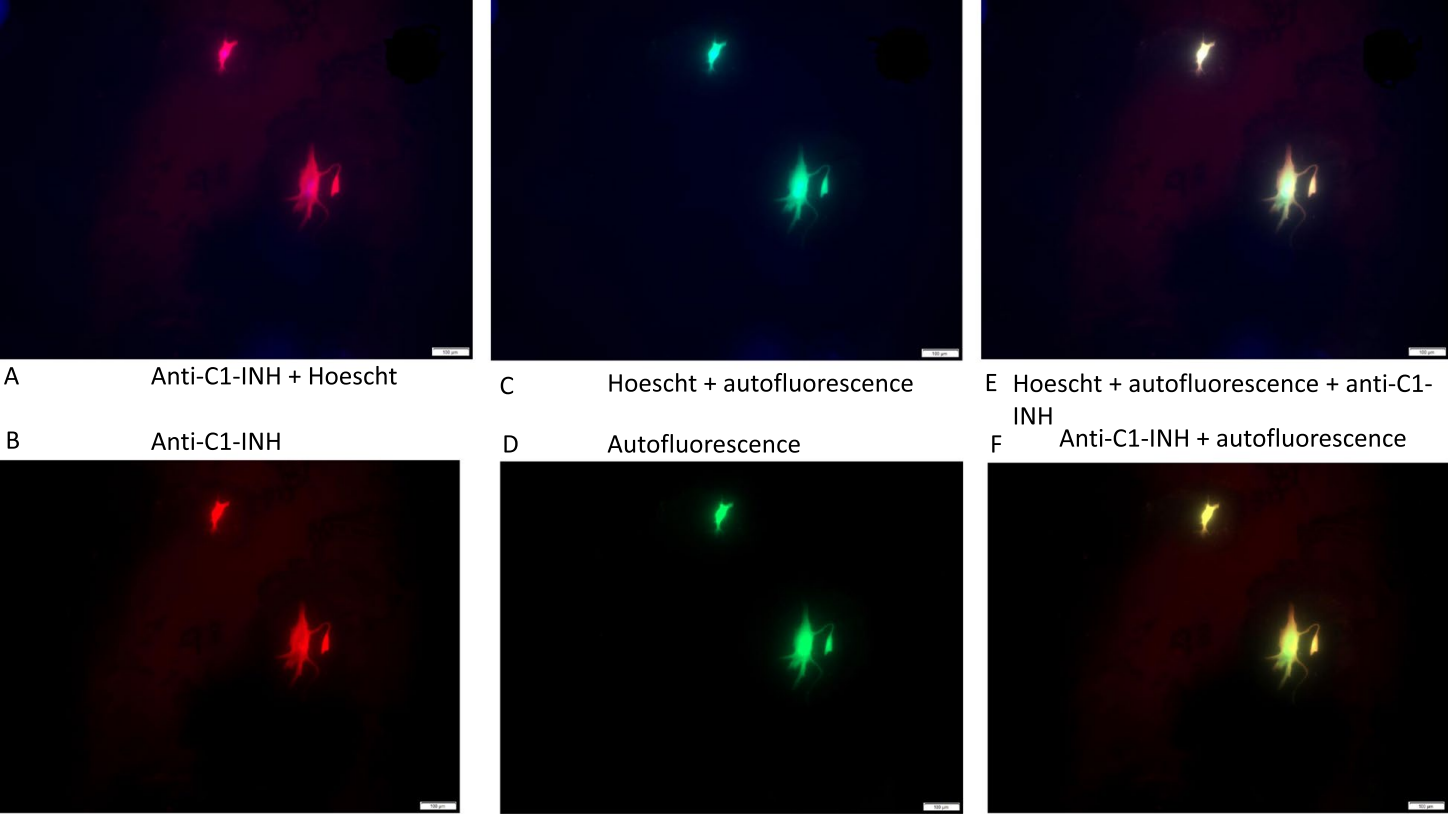
Anti-C1-INH expression (rabbit anti-rat Covance) demonstrated on NS1 cells in red. NS1 autofluorescence in green and nuclear staining with Hoechst. Scale bar 100 µm in figures **A**-**F**. **A** Strong anti-C1-INH staining in NS1 cells demonstrated with red. Nuclear staining with Hoescht. **B** Anti-C1-INH demonstrated with red. **C** Green autofluorescence of the NS1 cells and nuclear staining with Hoescht. **D** Green autofluorescence. **E** Green autofluorescence, Hoeschst staining and anti-C1-INH merged together, demonstrating distinct overlap. **F** Anti-C1-INH in red and green autofluorescence

C1-INH expression of the NS1 glioblastoma cells could be clearly demonstrated in vitro (Fig. 1a-b). Almost all NS1 cells displayed a distinct binding of anti-C1-INH. The NS1 cells also expressed GFP, as expected (Fig. 1c-d). GFP and anti-C1-INH staining correlated well (Fig. 1e-f).

### Increased survival in irradiated animals but no added effect of anti-C1-INH in animals with intracranial glioblastoma

**Fig. 2:**
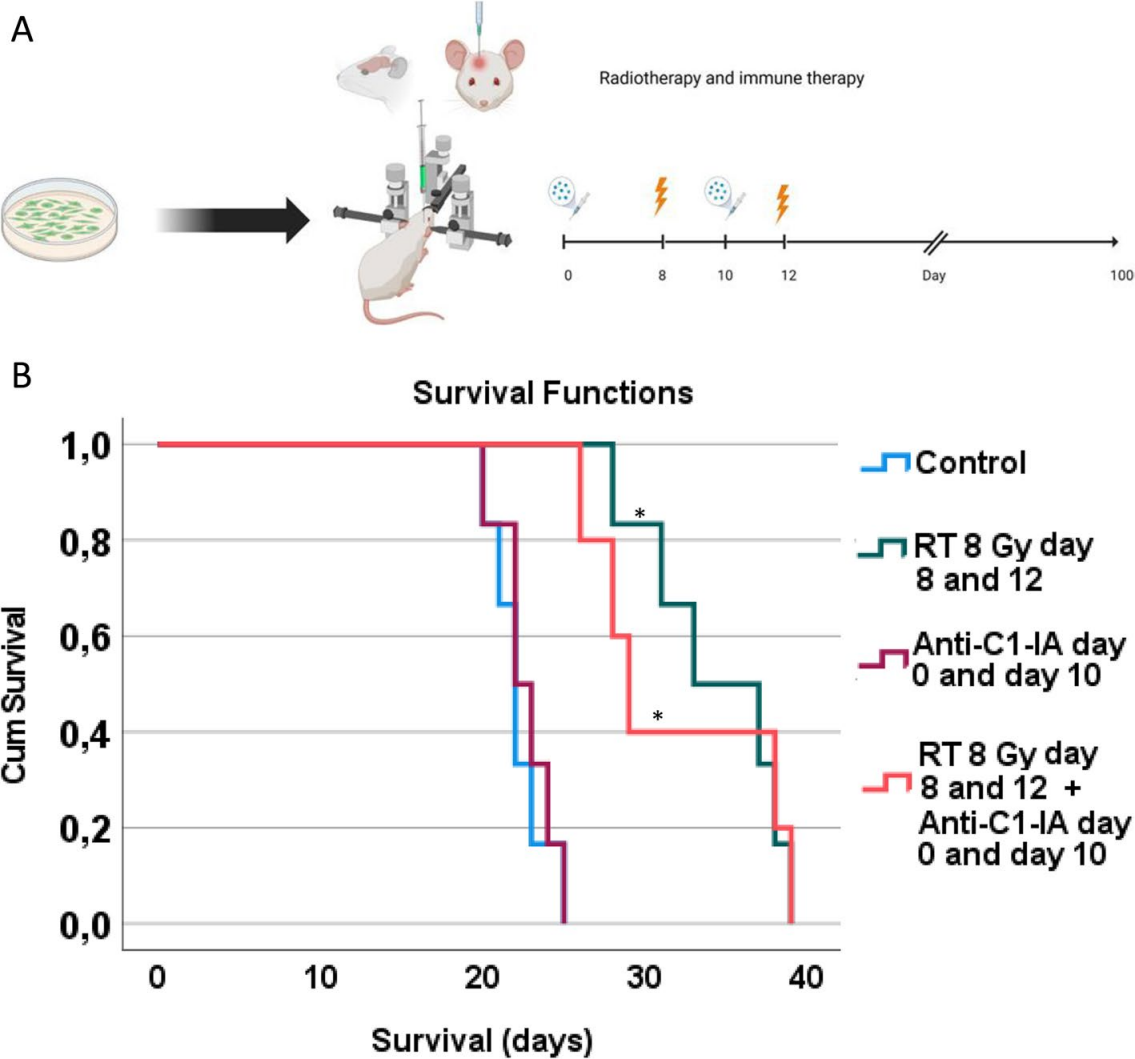
**A** Experimental setup in animals with intracranial tumors (image created with BioRender). **B** Increased survival was observed in animals treated with radiotherapy, whereas no additional effect of low-dose anti-C1-INH delivered intratumorally could be detected

The efficacy of treating animals with intracranial tumors was evaluated. 23 animals that had been inoculated with intracranial NS1 tumors were included. Animals were treated with irradiation and immunotherapy (as defined in Fig. 2a), and divided into four groups as follows: control animals with tumor inoculations but no further treatment (*n* = 6); animals treated with RT 8 Gy × 2 (*n* = 6); animals treated with intratumoral anti-C1-INH (*n* = 6) and animals treated with both RT 8 Gy × 2 + anti-C1-INH (*n* = 5). Survival was defined as days after tumor cell inoculation until criteria for euthanasia were fulfilled. Survival data was compared using Kruskal–Wallis test and Mann–Whitney test with post-hoc Bonferroni correction, even though it was normally distributed, but since only 5–6 samples were available per group.

Survival was significantly increased in animals treated with radiotherapy (RT 8 Gy × 2 versus control *p* = 0.007). Anti-C1-INH together with radiotherapy also increased survival significantly, but there was no synergistic effect of adding anti-C1-INH to radiotherapy (RT 8 Gy × 2 + anti-C1-INH versus control, *p* = 0.032; median survival RT 8 Gy × 2 = 35 days and median survival RT 8 Gy × 2 + anti-C1-INH = 29 days). There was no significant effect of anti-C1-INH as stand-alone therapy (anti-C1-INH versus control, *p* > 0.05) (Fig. 2b, Table 1). There was a power of 0.86 at detecting differences between groups at the 0.05 significance level. Increasing the concentration of intratumoral anti-C1-INH did not yield any increased effect compared to control animals delivered as single therapy (S1).

**Table 1:** Survival of animals treated with or without intratumoral anti-C1-INH in the intracranial setting, with or without radiotherapy

Treatment (number of animals per group)	Survival (median days ± SD)
Controls (*n* = 6)	22 ± 2
Anti-C1-INH intratumorally (*n* = 6)	23 ± 2
RT 8 Gy × 2 (*n* = 6)	35 ± 4
RT 8 G × 2 + anti-C1-INH intratumorally (*n* = 5)	29 ± 6

### Gene expression analysis

**Fig. 3:**
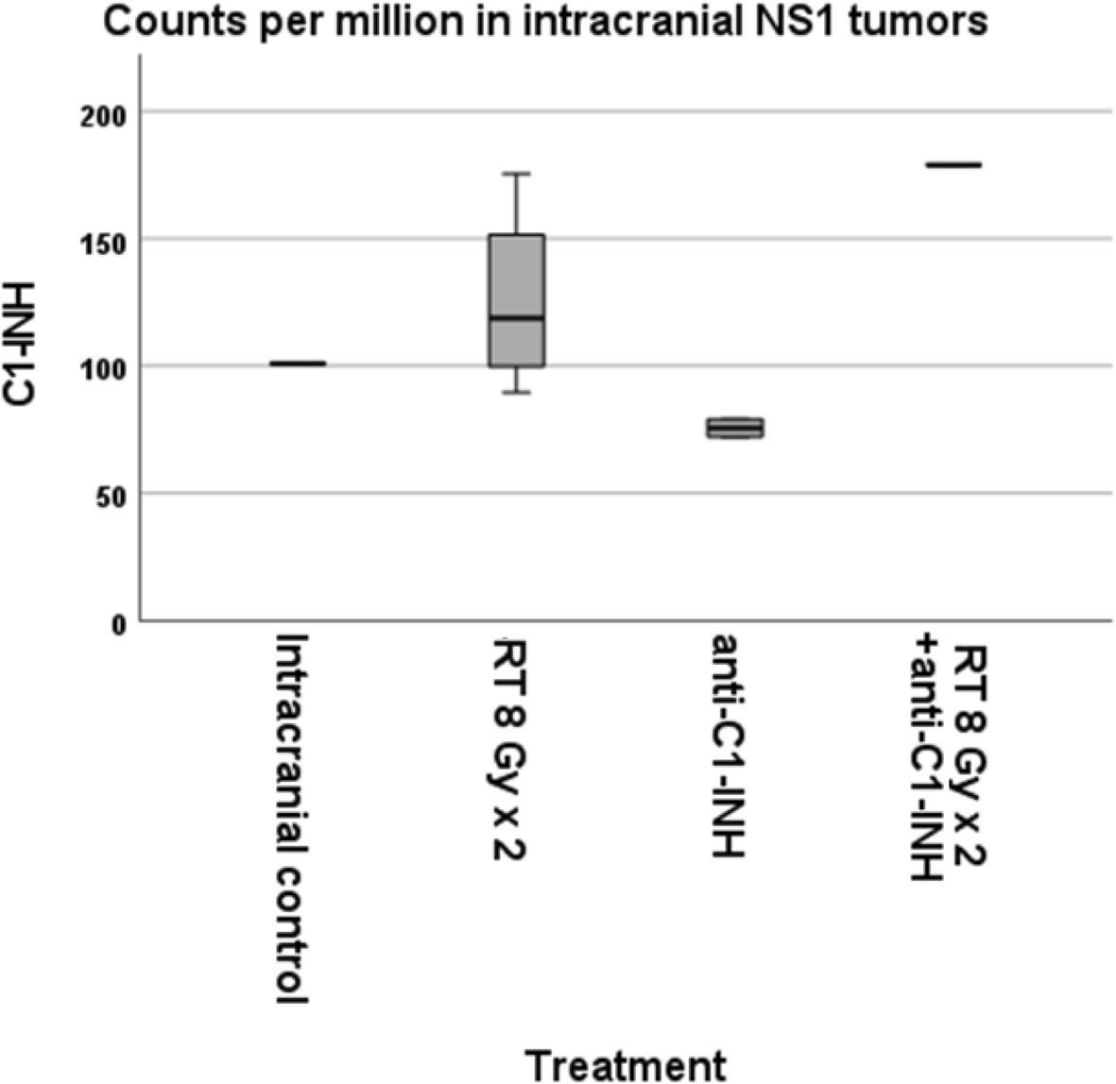
Expression of C1-INH in animals with intracranial tumors. The C1-INH expression was decreased in animals treated with anti-C1-INH compared to control, but it did not reach statistical significance. Adding radiotherapy increased the C1-INH expression, but also below limits for statistical significance. Hypothetically, the irradiation could sensitize the animals for anti-C1-INH treatment by increasing the C1-INH expression

Tumor tissue was harvested from animals with intracranial tumors. C1-INH was initially analyzed. The expression was reduced in animals treated with anti-C1-INH compared to control animals. C1-INH expression was increased in irradiated animals, although measurement did not meet statistical significance (Fig. 3).

The total list of differently expressed genes was explored in relation to factors associated with the classical complement pathway, including C1q, C1r, C1s, C1-INH, C2, C3, C4, C5, C7, C8, C9, CD55 and CD59. C1r, C1s, C2, C3, C4b, CD55 were found to differ between groups (Table 2). C1r, C1s, C2, C3, C4b were increased in animals treated with combined radiotherapy and anti-C1-INH compared to control tumor, but the down-stream components in the classical pathway were not increased. Also, CD55 was increased in the combined radiotherapy and anti-C1-INH group when compared to tumors from control animals. CD55 (decay-accelerating factor, DAF) is one of the regulators of the complement cascade [32]. CD55 is expressed on nearly all cells of the body and overexpressed on tumor cells [32].

**Table 2:** Differently expressed genes. RT = animals treated with RT 8 Gy × 2; RT + anti-C1-INH = animals treated with RT 8 Gy × 2 + anti-C1-INH; Anti-C1-INH = animals treated with anti-C1-INH; Control = animals inoculated with tumor cells but without no further treatment. The gene expression was increased in the first-mentioned group in the column “Group” if fold change > 0; and decreased if fold change was < 0; ie fold change > 0 comparing RT versus Control means that the expression was increased in irradiated animals compared to control animals

Gene name	Fold change (log2)	*p*-value (corrected for multiple comparisons)	Groups
C1r	2,565,566,234	4,27E-07	RT + anti-C1-INH versus Control
C1s	3,449,858,746	4,04E-09	RT + anti-C1-INH versus Control
C1s	2,684,553,496	1,99E-07	RT versus Control
C2	2,898,202,831	7,74E-08	RT + anti-C1-INH versus Control
C2	2,181,478,879	3,50E-06	RT versus Control
C3	-4,42,863,084	1,42E-05	Control versus Anti-C1-INH
C3	3,750,450,877	2,45E-04	RT + anti-C1-INH versus Control
C3	3,024,466,599	1,01E-03	RT versus Control
C4b	3,889,746,413	9,18E-09	RT + antiC1-INH versus Control
C4b	2,689,104,385	4,80E-06	RT versus Control
Cd55	2,270,818,549	9,66E-06	Control versus Anti-C1-INH
Cd55	-2,384,966,084	0,000,275,229	RT + anti-C1-INH versus Control

**Fig. 4:**
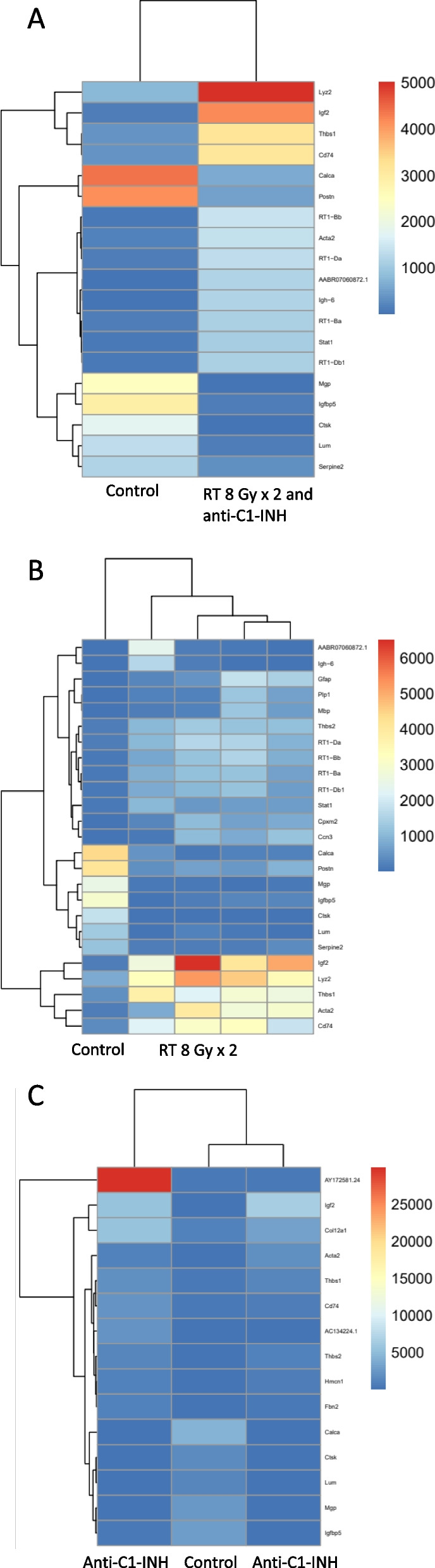
The genes with highest expression that were differently expressed between groups. **A** Comparing RT 8 Gy × 2 and anti-C1-INH to control. **B** Comparing RT 8 Gy × 2 to control. **C** Comparing anti-C1-INH to control

The highest expressed genes with statistically significant differences between the groups, all with > 1000 cpm, were analyzed in detail (Fig. 4a-c). Serpine2 was reduced in animals treated with RT and RT together with anti-C1-INH compared to control. High expression of Serpine2 has been connected to increased cellular migration and proliferation in squamous carcinoma, and it has also been suggested as a cancer-promoting factor that increases angiogenesis [33]. Serpine2 expression seems to vary across tumor stages and tissue types [34], and its exact role in glioblastoma is not yet known, but it seems to be abundant in glioblastoma, whereas its expression is very low in meningioma [34]. Igf2 was increased after treatment with anti-C1-INH, and combined RT and anti-C1-INH compared to control. Igf2 has been demonstrated in the brain of rodents, possibly increased in hippocampus and neural stem cells [35]. Thbs1 was increased in all treated groups compared to control. Upregulation of Thbs1 has been shown to be increased in glioma of higher grade and has been associated with poor prognosis [36]. Possibly, the upregulation of ThBs1 seen in the specimens of treated glioblastomas is an important driver of tumor progression despite treatment, and an interesting target for future additional therapies.

### Increased survival after combined treatment with anti-C1-INH and irradiation in animals with subcutaneous glioblastoma

From the intracranial experiments, we could conclude that treatment with RT combined with anti-C1-INH altered many of the upstream genes of the classical pathway of the complement system. However, there were no significant alterations of the downstream components, and no synergistic or additional effect of adding anti-C1-INH to RT when it came to survival from tumor inoculation prior to presentation of symptoms related to tumor growth.

In the next step, we wanted to explore if efficacy could be increased if tumors were located subcutaneously instead of intracranially. With subcutaneous tumors, the dose of anti-C1-INH could be substantially increased in accordance to animal ethics regulations. Furthermore, the blood–brain barrier (BBB) is not present, possibly increasing the efficacy of immune therapy.

**Fig. 5:**
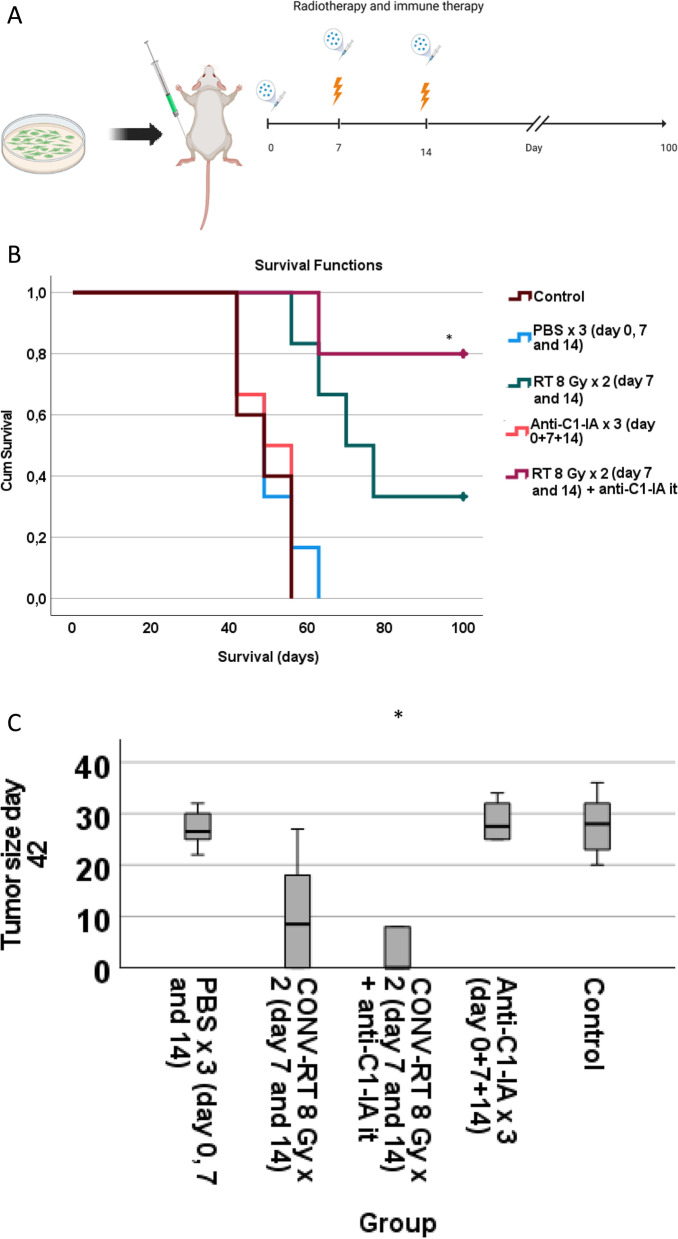
**A** Experimental setup in animals with subcutaneous tumors (image created with BioRender). **B** In the subcutaneous setting, treatment with combined RT + anti-C1-INH resulted in significantly prolonged survival and long-term anti-tumor control compared to non-treated control animals. **C** Combined treatment with RT + anti-C1-INH also significantly reduced tumor size, as seen on day 42 after tumor cell inoculations compared to non-treated control animals; the last day when all animals were still represented in each group. After day 42, animals had to be euthanized due to the size of the subcutaneous tumors exceeding 30 mm as defined in ethics regulations

The same glioblastoma cell line was used. 28 animals were included in the study of subcutaneous tumors (Fig. 5a; Table 3); control animals inoculated with tumor cells but without any further treatment (*n* = 5); animals treated with RT at 8 Gy × 2 (*n* = 6); animals treated with intratumoral anti-C1-INH (*n* = 6); animals treated with RT at 8 Gy × 2 + anti-C1-INH (*n* = 5); animals treated with PBS intratumorally (*n* = 6). Intratumoral PBS was added, since the antibodies were dissolved in PBS, and we wanted to rule out that just injecting an extra volume of PBS would disrupt the tumor growth. Survival data was calculated as days from inoculation until euthanasia, and was not normally distributed. Animals that euthanized prior to the end of the study at day 100 were done so due to tumor growth exceeding the maximally allowed diameter of 30 mm.

**Table 3:** Survival of animals with subcutaneous tumors

Treatment (number of animals per group)	Survival (median days ± SD)
Controls (*n* = 5)	49 ± 7
PBS intratumorally (*n* = 6)	49 ± 8
Anti-C1-INH intratumorally (*n* = 6)	53 ± 7
RT 8 Gy × 2 (*n* = 6)	74 ± 19
RT 8 G × 2 + anti-C1-INH intratumorally (*n* = 5)	100 ± 17

Survival differed significantly between groups (Kruskal–Wallis *p* < 0.001). Compared to control animals, survival was significantly increased in animals treated with RT + anti-C1-INH versus control animals (Mann–Whitney U-test with post-hoc Bonferroni correction *p* = 0.024) whereas the other groups did not differ significantly from the control animals (Fig. 5b; Table 3). Animals treated with combined RT + anti-C1-INH had 60% long-term survivors, still alive at day 100 after tumor inoculations (Table 4). The estimated power to detect a difference at the 0.05 level was 0.92.

**Table 4:** Animals with long-term tumor control did not display any signs of tumor growth 100 days after tumor cell inoculations

*Animals with no detectable tumor on day 100 in subcutaneous study*	Number (%)
Controls	0 (0)
PBS intratumorally	0 (0)
Anti-C1-INH intratumorally	0 (0)
RT 8 Gy × 2	2 (33.3)
RT 8 G × 2 + anti-C1-INH intratumorally	3 (60)

Next, we compared tumor size between the treatment groups, as long as all animals were still alive in all groups. At day 42 after initiation of the experiment, the first animal was euthanized due to tumor growth. Tumor size differed significantly between the groups (Kruskal–Wallis *p* = 0.002). Compared to control animals, tumor size was significantly reduced in animals treated with RT + anti-C1-INH (Mann–Whitney U-test with post-hoc Bonferroni correction *p* = 0.042), whereas the other groups did not differ significantly from the control animals (Fig. 5c).

**Fig. 6:**
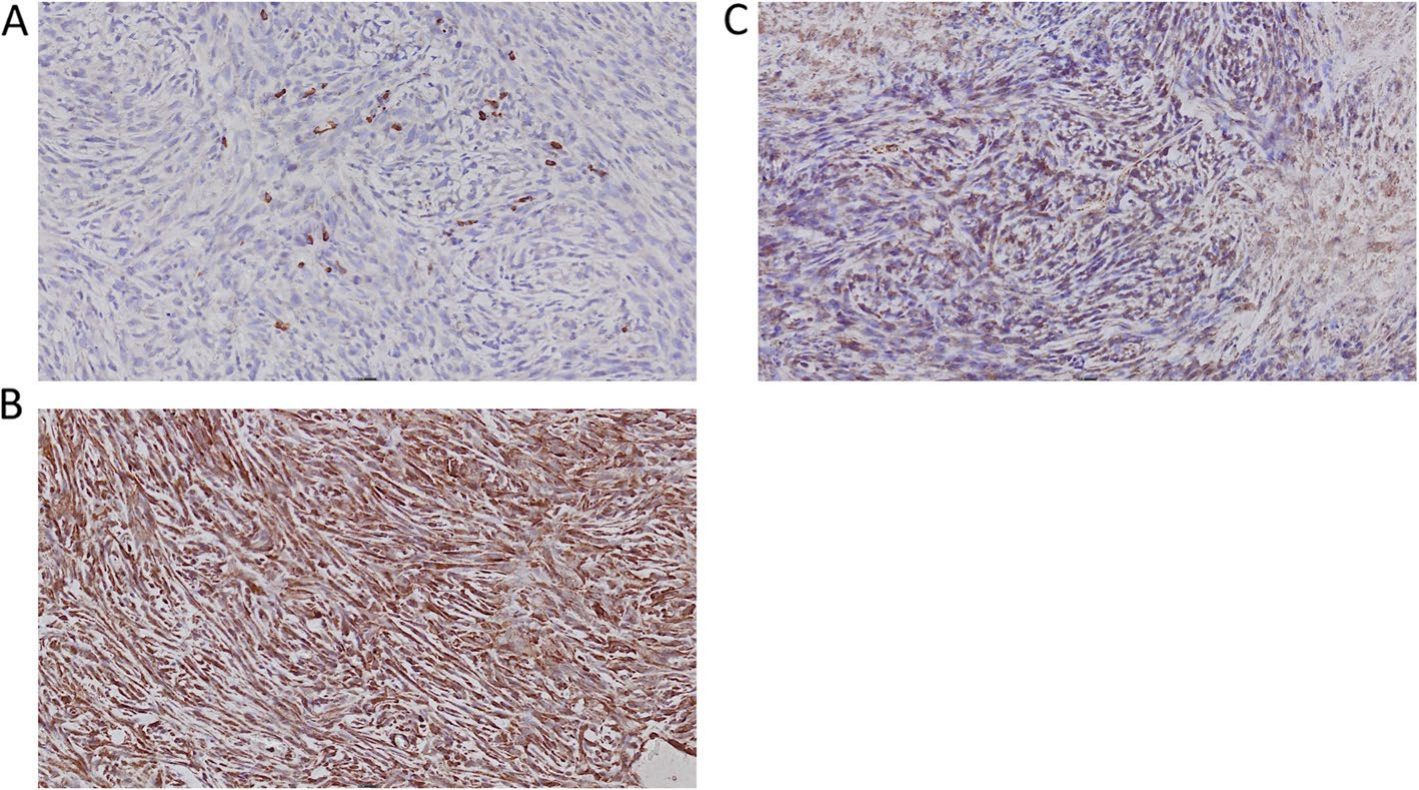
Immunohistochemistry of subcutaneous tumor from an animal that deceased early (day 56), treated with RT 8 Gy × 2. **A** CD4. **B** CD8. **C** FOXP3

**Fig. 7:**
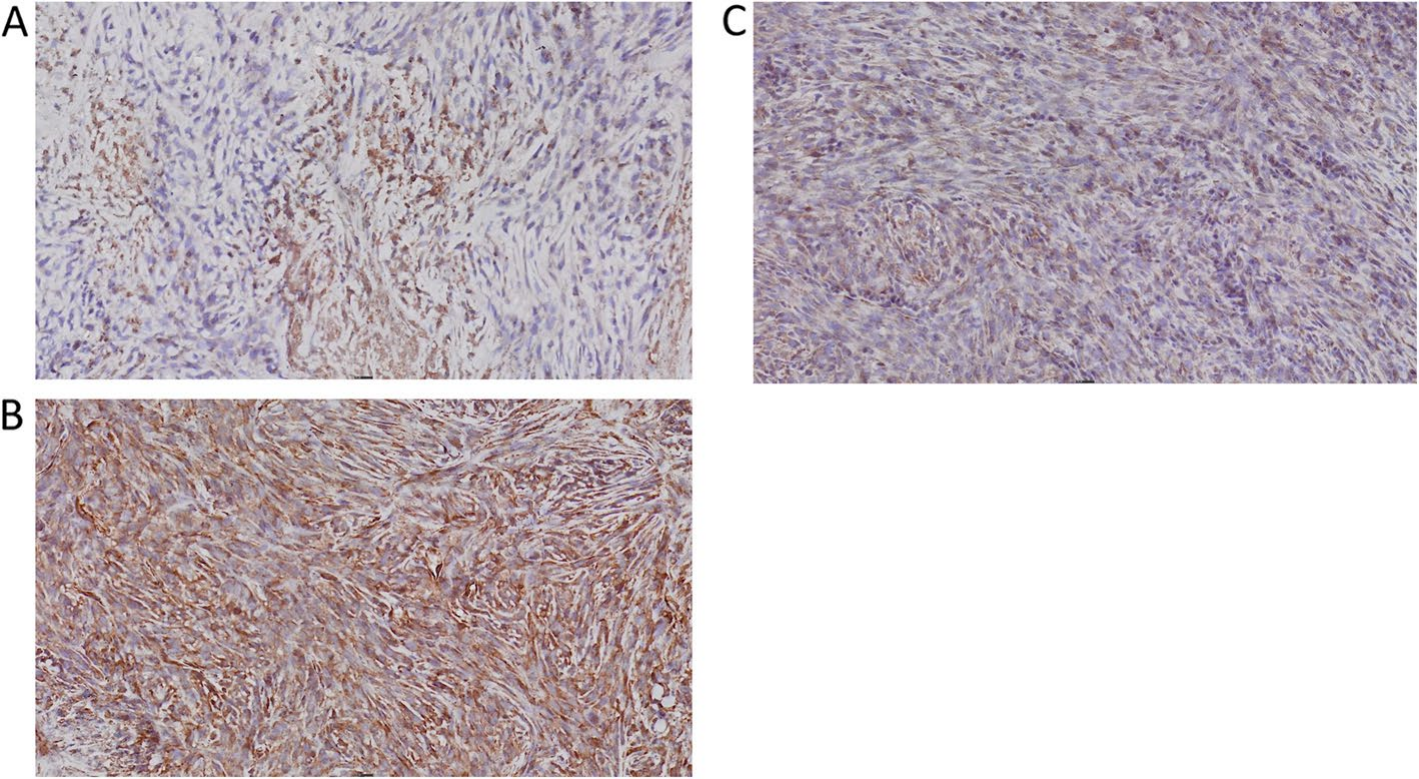
Immunohistochemistry of subcutaneous tumor from a control animal that deceased the same day as the irradiated animal presented in Fig. 6 (day 56). **A** CD4. **B** CD8. **C** FOXP3

The immunohistochemical expression of CD8, CD4 and FOXP3 positive cells was evaluated in tumor tissue samples in relation to radiological treatment. We compared expression in animals that deceased on the same day (identical survival time) after tumor cell inoculations due to large tumors (eight weeks after tumor cell inoculations, six weeks after finished irradiation). Both irradiated and control animals generally exhibited relatively intense CD8 positivity, except necrotic areas, where there was no specific staining (Figs. 6 and 7), whereas staining with CD4 and FOXP3 was detectable, but sparse in comparison. From an immunohistochemical point of view, no pattern could be defined that clearly distinguished the control tissue from the irradiated tissue.

## Discussion

In the present study we could demonstrate a long-term anti-tumor effect in animals treated with combined radio-immunotherapy against glioblastoma in the subcutaneous setting, compared to the effect of each treatment as stand-alone therapy. As single treatments, the radiotherapy and immunotherapy were delivered at supposedly sub-therapeutic levels, in order to explore possible synergistic effects of the combined treatment. A major strength with the present study is that it was done in a fully immunocompetent animal model. In the present study we decreased the field size of irradiation to 1 × 1 cm compared to 2 × 2 cm in animals with subcutaneous tumors in a previous study [28] and doses of anti-C1-INH were reduced compared to previous protocols with anti-C1-INH treatment [12]. Long-term tumor control was, within the parameters described, only achieved in animals that had received a combined immuno-radiotherapy. Trying to treat intracranial tumors was not found equally effective. Possibly, the lower doses of anti-C1-INH administered intracranially compared to the subcutaneous doses were too low to generate a strong immunological response. Regarding intratumoral delivery intracranially, we were limited by the amounts allowed from an animal ethics perspective. With systemic intravenous administration of anti-C1-INH, there would possibly be a need to initially neutralize circulating anti-C1-INH, before effects on the tumor could be achieved, which might increase the risk of side effects. However, the efficacy of intravenous or even selective intra-arterial anti-C1-INH administration could be explored further in future pre-clinical studies.

From the gene analyses we could demonstrate down-regulation of C1-INH as a result of the anti-C1-INH treatment intracranially, but it did not meet statistical significance. One limitation with the gene expression analysis was that we could only include samples with sufficient RNA quality, and the tumors from all animals did not pass these quality checks. Thus, potentially, effects might have been missed due to the few samples analyzed. Still, up-stream mediators of the classical pathway of the complement system were increased in animals with intracranial tumors that had received treatment compared to the control animals. This was an interesting observation, since no significant difference, neither increase nor decrease, was observed in the down-stream components of the classical complement cascade. In relation to tumor treatment, complement seems to have opposing roles at different concentrations [32]. Transfecting tumor cells with C5a, could lead to both increased and decreased tumor growth, depending on C5a expression. C5a is an important immune cell mediator, that interacts with the C5a receptor, leading to vasodilatation and entry of immune cells [32]. In experiments, C5a overexpression led to increased infiltration of NK cells and macrophages into the tumor tissue, and reduced tumor growth [32]. In the present study, we demonstrated increased expression of C3 in animals treated with anti-C1-INH, compared to control tumor tissue, whereas C5 was not significantly altered. Furthermore, survival was not increased in animals with intracranial tumor treated solely with anti-C1-INH, indicating that the increase of C3 was not enough, and possibly, downstream activators of the complement system were not reached. Apart from C1-INH, the complement system is also regulated by several other proteins. One important regulator is CD55, that accelerates dissolution of C3 and C5 convertase [32]. Interestingly, CD55 expression was reduced in animals treated with anti-C1-INH and combined irradiation and anti-C1-INH.

The blood–brain barrier (BBB) is known to render the brain and its tumors an immune privileged site [37]. Even though the BBB is partially disrupted in glioblastoma, tumors cells are also hiding behind an intact barrier. Focused ultrasound (FUS) is one technique that could be applied in order to increase the BBB passage. It has been demonstrated that in animals receiving radiosurgery, FUS can open the BBB and increase delivery of systemic therapy [38]. No morbidity or mortality could be defined in relation to FUS in that study. Moreover, neurotrophic compounds have also been successfully delivered across the BBB with MRI guided FUS, such as glial cell line-derived neurotrophic factor [39]. Based upon the results of increased efficacy of combined immunotherapy and radiotherapy in our subcutaneous setting, but not in the intracranial setting, we hypothesize that accomplishing BBB opening with methods such as FUS might be beneficial also by applying concomitant radio-immunotherapy.

Timing of RT induced immunological effects could also play and important and not yet fully understood role. According to studies in other cancer models, the timing of RT and immune checkpoint blockade seems to matter; for example, PDL1 has been shown to be upregulated 24–96 h post RT; and delivery of PDL1 blockade 7 days after RT did not yield any survival benefit, as compared to 1 or 5 days in colon carcinoma [40]. The relation between timing and C1-INH response to radiotherapy is not known yet, but based upon other studies such as [40], it might affect the outcome as well.

## Conclusions

In the present study we could demonstrate that anti-C1-INH treatment combined with radiotherapy increased survival in animals with subcutaneous glioblastoma. In the intracranial setting, we could not demonstrate any effect of anti-C1-INH treatment, which could be due to several factors. The most important factor may likely be too low doses and possibly the role of the BBB.

## Supplementary Information

**Additional file 1.**

## Data Availability

Results of gene datasets analyzed during the current study have been submitted to the Array Express repository (accession number [E-MTAB-12296]). Other data generated in the current study is available upon reasonable request upon contact with the corresponding author.
