# Supplementary material for: Combined anti-C1-INH and radiotherapy against glioblastoma
Source: BMC Cancer. 2023 Jan 30;23:106. doi: 10.1186/s12885-023-10583-1 (PMC9887755; doi:10.1186/s12885-023-10583-1)
Supplement: Supplementary file 1 — Additional file 1. [file 12885_2023_10583_MOESM1_ESM.docx]

**Supplementary material S1**

In order to evaluate if increased concentrations of anti-C1-INH intratumorally would yield any survival benefit in the intracranial situation, anti-C1-INH was delivered intratumorally at day 0 and 12, but at increased concentrations. For comparison, another group of animals was injected with intratumoral PBS as well, in order to define if the medium that the antibodies were dissolved in would lead to any reaction. Animals were treated with 0.5 ul concentrated anti-C1-IA (50 mg/ml) (Covance) day 0 and 12, or intratumoral PBS, or were untreated control animals.

Eighteen animals were included (n=6 controls; n=6 PBS controls; n=6 treated with concentrated anti-C1-INH). Survival was monitored, and was not normally distributed. There was no statistical significance between the groups (Kruskal-Wallis p > 0.05) (Figure S1).


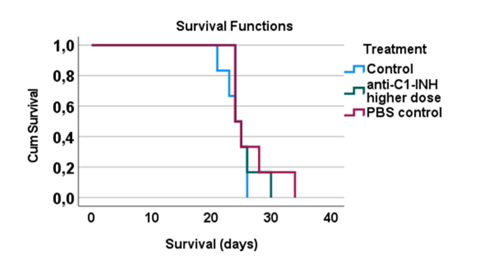


**Figure S1**

Increasing the dose of intratumoral anti-C1-INH did not increase survival compared to control animals inoculated with tumors but without any further treatment, or those treated with the medium PBS intratumorally.
